# Perforated Jejunal Pseudodiverticulum Managed With Limited Segmental Resection: A Case Report

**DOI:** 10.7759/cureus.105339

**Published:** 2026-03-16

**Authors:** Luis Carlos Lozano-Carrillo, Bernardo Alfonso Fernández-Reyes, Stephie Oyervides-Fuentes, Rodrigo Enrique Elizondo-Omaña, Alejandro Quiroga-Garza

**Affiliations:** 1 Department of Human Anatomy, Clinical and Surgical Research Group (GICQx), Universidad Autónoma de Nuevo León, School of Medicine, Monterrey, MEX; 2 General Surgery Division, Instituto Mexicano del Seguro Social, Hospital de Traumatologia y Ortopedia #21, Monterrey, MEX

**Keywords:** case report, diverticulitis, jejunal diverticula, perforation, pseudodiverticula

## Abstract

Jejunal diverticulitis (JD) is a rare disease with significant clinical relevance, as it often causes intense pain in the epigastrium that radiates to the left flank, accompanied by hemodynamic instability and leukocytosis. Diagnosis must be complemented with clinical and imaging studies.

A 72-year-old woman presented with abdominal pain of colic type in the epigastrium and left flank for 12 hours. The computed tomography scan showed two diverticula in the jejunum, the first with localized pneumoperitoneum and signs of perforation, the second with no signs of an inflammatory process. An exploratory laparotomy was indicated, identifying perforation of a diverticulum. Jejunal resection and primary anastomosis were performed. The postoperative period was uneventful, with no complications at 30-day follow-up.

JD is an uncommon pathology with high morbidity due to its diagnostic difficulty. There are no established guidelines for the treatment of this pathology, however medical management with a broad-spectrum antibiotic approach, accompanied by intravenous fluid resuscitation, has proven effective in stabilizing vital signs and laboratory parameters of patients, which favors the clinical status and outcome of subsequent surgery. Upon perforation, definitive surgical management is needed.

## Introduction

Small-intestinal diverticula are false diverticula (pseudodiverticula) that do not involve the full thickness of the intestinal wall. Although diverticula are more common in the colon, small-intestinal diverticula are rare; duodenal diverticula are the most frequent (22%), followed by jejunal diverticula (0.06-2.3%). Despite their rarity, reported mortality can reach up to 30%, underscoring the importance of early diagnosis and timely management [1-5]. Diagnosis is challenging because presenting symptoms are non-specific (abdominal pain, nausea, vomiting, bloating, diarrhea, flatulence), and complications (perforation, abscess, hemorrhage, obstruction) develop in roughly 20% of patients [5,6]. Jejunal diverticulosis refers to the presence of mucosal-submucosal outpouchings (pseudodiverticula) that likely arise by herniation through weak points in the muscularis propria along the mesenteric border under increased intraluminal pressure, whereas diverticulitis denotes inflammation and/or infection of these diverticula [6].

For perforated jejunal diverticulitis (JD), conventional management remains surgical resection of the affected segment. Nonetheless, inpatient conservative therapy with IV fluids and broad-spectrum antibiotics has been reported in selected patients (by analogy with colonic diverticulitis) particularly in uncomplicated presentations, where diet may be restricted initially and advanced as symptoms improve and antibiotics used selectively (e.g., comorbid/frail patients, refractory symptoms or vomiting, C-reactive protein (CRP) >140 mg/L, WBC >15 × 10⁹/L, or CT showing a fluid collection or a longer inflamed segment). Case reports suggest these regimens require close inpatient monitoring over hours to days, ranging from clinical improvement within 48 hours on IV piperacillin/tazobactam to prolonged therapy with nasogastric decompression and IV meropenem with discharge on day 20 after percutaneous drainage. In complicated disease (abscess, perforation, sepsis, or obstruction), antibiotics are mandatory and operative source control should not be delayed when perforation is demonstrated [6,7]. 

We present the case of a woman with two jejunal diverticula, one demonstrating radiologic signs of contained perforation, reported in accordance with the Surgical Case Report (SCARE) guidelines.

## Case presentation

A 72 -year-old Caucasian female patient (BMI 27.6 kg/m2) presents to the emergency room due to abdominal pain. She has a history of uterine fibroids treated with hysterectomy 32 years ago, breast cancer treated with right radical mastectomy, with adjuvant chemotherapy and radiotherapy 16 years ago, liver metastasis treated with radical hepatectomy, post-incisional hernia treated with conventional hernioplasty 14 years ago, chronic hypothyroidism, and dyslipidemia under medical treatment. She refers a 12-hour history of acute abdominal pain, localized in the left flank. The pain was reported as colic-type, of moderate intensity, with radiation to the epigastrium and left iliac fossa, accompanied by nausea without vomiting.

On arrival at the hospital, the patient had a heart rate of 97 beats per minute, temperature of 36.5ºC, and systolic hypertension of 161/76 mmHg. On physical examination, the patient had increased epigastrium volume and tenderness in the left flank and iliac fossa. Initial laboratory and venous blood gas evaluation demonstrated marked leukocytosis with neutrophilia (WBC 19.25 K/µL; neutrophils 80%) and an elevated CRP level (5.56 mg/dL), while liver and renal function parameters were within reference ranges (Table 1).

**Table 1:** Admission laboratory results

Parameter	Value	Reference range
White blood cell count (WBC)	19.25 K/µL	3.8–11.6 K/µL
Neutrophils	80%	38.4–74.6%
C-reactive protein (CRP)	5.56 mg/dL	<0.5 mg/dL
Total bilirubin	0.21 mg/dL	<1.2 mg/dL
Direct bilirubin	0.12 mg/dL	0.09–0.30 mg/dL
Blood urea nitrogen (BUN)	16.4 mg/dL	6–20 mg/dL
Glucose	117 mg/dL	55–99 mg/dL
Creatinine	0.69 mg/dL	0.70–1.20 mg/dL
Uric acid	3.8 mg/dL	3.4–7.0 mg/dL
Lactate	2.2 mmol/L	0.5–2.2 mmol/L
pCO₂	32 mmHg	35–45 mmHg
pO₂	32 mmHg	30–40 mmHg

An abdominal radiograph was obtained and was inconclusive. She was admitted to the General Surgery service, and medical management was initiated on admission with intravenous resuscitation using Hartmann’s solution (1,000 mL as a continuous infusion over eight hours), along with ceftriaxone (1 g IV every 12 hours) and metronidazole (500 mg IV every eight hours), plus antiemetics and nonsteroidal anti-inflammatory drugs (NSAIDs).

**Figure 1:**
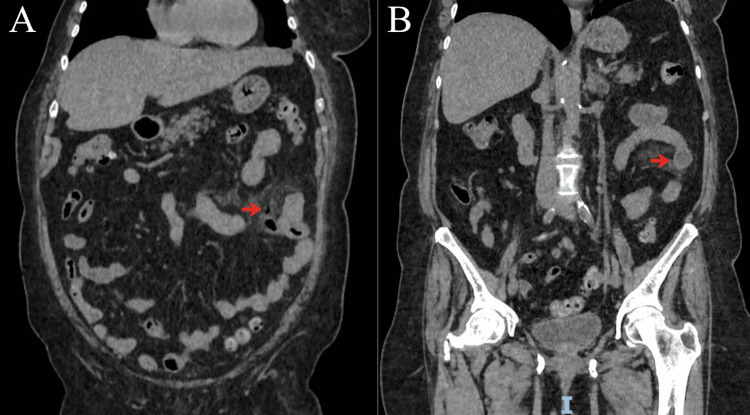
Contrast-enhanced abdominal CT, coronal reformations. A) Perforated jejunal diverticulum on the mesenteric border with peridiverticular fat stranding and a tiny focus of localized extraluminal air adjacent to the sac (arrow), consistent with contained perforation. B) Non-perforated proximal jejunal diverticulum with mural thickening (arrow) and no extraluminal air or upstream obstruction.

Initially, the patient responded favorably to medical management, with stabilization of vital signs within 12 hours (blood pressure 103/64 mmHg, heart rate 83 bpm, respiratory rate 18 breaths/min, temperature 36.5°C). The following day, contrast-enhanced abdominal CT was performed and demonstrated two diverticula in the proximal left jejunum measuring 1.8 cm and 2.0 cm in diameter, respectively (Figure 1).

Adjacent to the proximal diverticulum, there was peridiverticular fat stranding and a small, localized focus of extraluminal air consistent with localized pneumoperitoneum/contained perforation, without diffuse free intraperitoneal air. No peritoneal fluid, abscess formation, or reactive mesenteric lymphadenopathy was identified. The patient remained hemodynamically stable; however, given the radiologic evidence of contained diverticular perforation (localized pneumoperitoneum) and persistent left flank and left iliac fossa tenderness, she was scheduled for exploratory laparotomy the same day.

**Figure 2:**
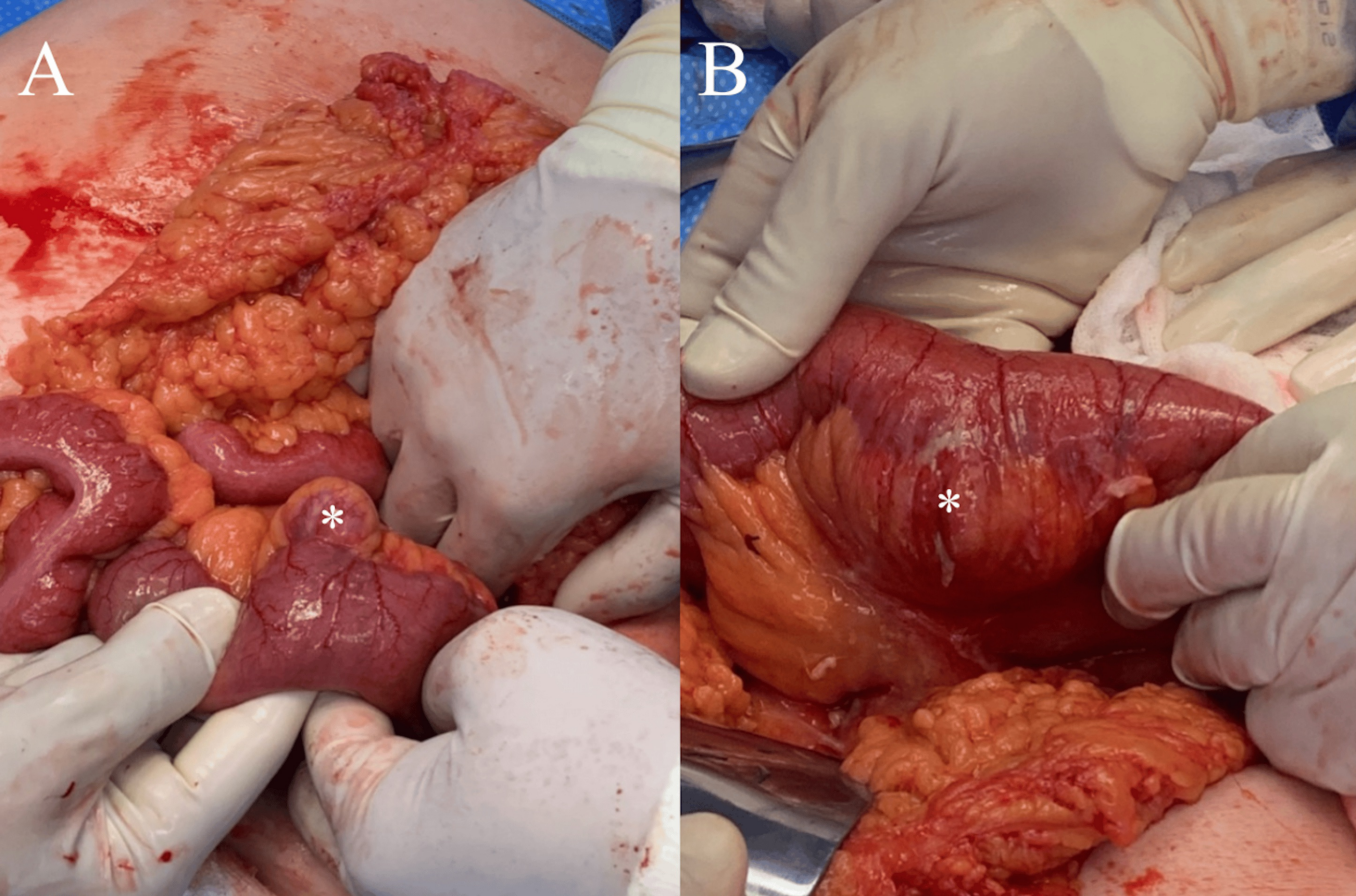
Gross specimen of jejunal segment containing the diverticulosis. A) Diverticula with thickened wall. B) Diverticula with perforation.

In the exploratory laparotomy, intestinal adhesions (Zuhlke IV) and the two diverticula previously mentioned in the CT were found. The first diverticulum was located 70 cm from the ligament of Treitz with a thickened wall and perforation, while the second diverticulum was located at 80 cm, which did not present an inflammatory process. Both were in the mesenteric side (Figure 2). 

Adherenciolysis was made, and a functional side-to-side stapled anatomosis was performed, with a 75 mm manual stapler. Approximately 20 cm of small bowel was resected, which contained the two diverticula. A Blake 19 French was placed in the left flank, and the abdominal wall was closed. The patient had a good postoperative result with clinical improvement in the following days. She was discharged with a soft, low-fat diet on the fifth postoperative day. Drain was removed on the 10th postoperative day.

**Figure 3:**
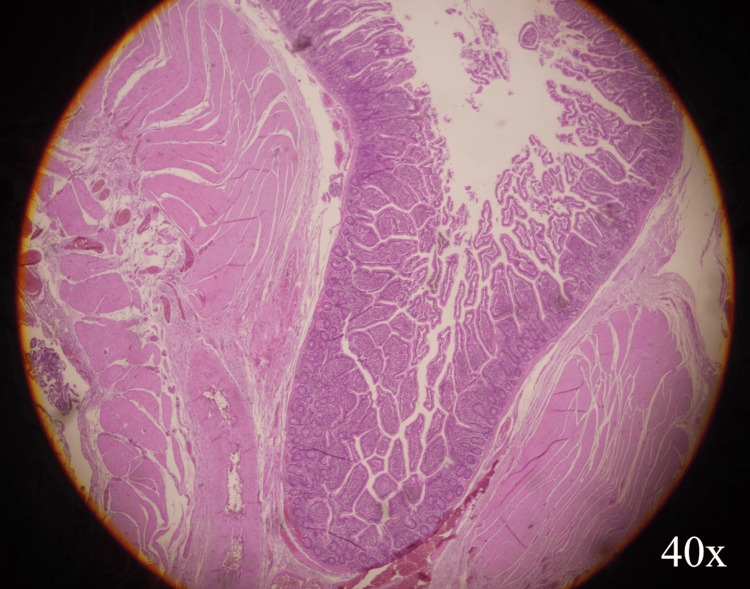
Jejunal pseudodiverticulum (H&E), original magnification ×40. Herniation of mucosa and submucosa through a focal defect of the muscularis propria at the diverticular neck (arrow), consistent with a false (pseudodiverticulum); background mucosa is preserved.

Follow-up in the outpatient clinic up to a month after postoperative day was uneventful. Histopathological examination of the resected specimen showed two mucosal invaginations that penetrate the muscular wall, corresponding to pseudodiverticula (Figure 3). The largest of these was perforated and surrounded by abundant acute inflammatory infiltrate, extensive fibrin deposits, and areas of necrosis.

## Discussion

This case describes a successful management of JD, diagnosed with CT. Surgical management was required due to complicated disease at diagnosis, and the postoperative course was uneventful. Notably, our case contributes to the existing literature by documenting multifocal jejunal pseudodiverticulosis with two proximal mesenteric-side diverticula, in which CT demonstrated contained perforation of one diverticulum while the second showed no inflammatory changes, a finding subsequently confirmed intraoperatively. This clinicoradiologic correlation supported a limited segmental jejunal resection encompassing both diverticula with primary anastomosis, achieving definitive source control while preserving bowel length.

Small-bowel diverticulosis has been recognized for centuries, and JD (although uncommon) is predominantly reported in older adults, with a peak incidence between 60 and 70 years [8-10]. Small bowel diverticula involve only the mucosa and submucosa layers; they are most frequently along the mesenteric border [11] as in this case. Most of jejunal diverticulosis cases are discovered incidentally, 10-30% become clinically apparent due to complications such as bleeding, obstruction, perforation, or diverticulitis [11].

The etiopathogenesis of jejunal diverticula remains incompletely understood. Most lesions are pseudodiverticula formed by herniation of mucosa and submucosa through weak points in the muscularis propria, typically along the mesenteric border where mesenteric vessels penetrate the bowel wall. A leading hypothesis is that abnormal intestinal motility and increased intraluminal pressure promote this herniation. Visceral neuropathy associated with connective tissue disorders has been reported as a potential contributor by increasing intraluminal pressure, thereby predisposing to diverticulosis [6].

A thorough evaluation accompanied by laboratory and imaging studies is important. An X-ray can detect the presence of pneumoperitoneum and help in the diagnosis. Abdominal CT is the most effective imaging technique for detecting this condition [12]. Identifying the corresponding site of perforation by observing inflammatory changes in the fat and localized pneumoperitoneum [13]. In mesenteric side diverticula, other modalities such as capsule endoscopy, double-balooon enteroscopy, and upper gastrointestinal studies may be useful for diagnosis [6,12,14]. They have limited application in emergency settings.

Surgery in older patients is associated with higher perioperative risk. Therefore, initial conservative management with broad-spectrum antibiotics, intravenous resuscitation, and bowel rest may be considered in carefully selected uncomplicated cases, as previously described [5,6,13]. However, this approach requires close monitoring because JD can progress to clinically significant complications, including gastrointestinal bleeding, perforation, peritonitis, localized abscess, adhesions/obstruction, and fistula formation [5,6,13]. Accordingly, failure to improve within 48-72 hours, or any radiologic/clinical evidence of perforation or sepsis, should prompt escalation to operative management to achieve definitive source control [13].

After observing perforation and inflammatory changes in imaging studies, the standard treatment is resection of the affected segments with primary anastomosis [12,13]. Different from Zafouri et al. [15], who reported a case of an elderly woman with an uncomplicated JD (no pneumoperitoneum/collection) with a giant single diverticulum 10 cm from Treitz; she improved on antibiotics and underwent elective diverticulectomy two weeks later, and they recommend diverticulectomy in case of diverticula near the duodeno-jejunal flexure to prevent anastomotic complications. Harbi et al. [16] recommend avoiding diverticulectomy and inversion in case of perforated diverticulum, due to high failure rate and increased mortality. 

Other surgical approaches have been proposed, such as the laparoscopic approach, which has been shown to be safe and feasible. Most prior case reports describe an upfront open approach or a diagnostic lap converted to open surgery [17]. The decision is based on the surgeon's expertise and patient characteristics. These minimally invasive approaches have also demonstrated a satisfactory postoperative outcome [18,19]. In our case, we opted for a laparotomy due to the surgical history of the patient.

## Conclusions

Although uncommon, JD is diagnostically challenging and has been associated with mortality rates of approximately 20-30% in complicated cases, largely attributed to delayed diagnosis. In the absence of robust guidelines, management should be individualized and driven by CT findings and patient factors. Early medical optimization (IV fluid resuscitation, analgesia, and appropriate broad-spectrum antibiotics) can stabilize patients and improve operative readiness. Nevertheless, when imaging demonstrates perforation with active inflammation, definitive surgical management with limited segmental resection and primary anastomosis remains the standard. A coordinated medical-surgical approach in the first hours of care is pivotal to favorable outcomes.
